# An unusual case of dysuria, pollakisuria, and eosinophilia: Questions

**DOI:** 10.1007/s00467-021-05128-2

**Published:** 2021-11-18

**Authors:** Fabian Eibensteiner, Ursula Tonnhofer, Alexander Springer, Hubert Kogler, Leila Ronceray, Azadeh Hojreh, Christoph Aufricht, Krisztina Rusai

**Affiliations:** 1 Division of Pediatric Nephrology and Gastroenterology, Comprehensive Center for Pediatrics, Medical University of Vienna, Waehringer Guertel 18-20, 1090 Vienna, Austria; 2 Division of Pediatric Surgery, Comprehensive Center for Pediatrics, Medical University of Vienna, Waehringer Guertel 18-20, 1090 Vienna, Austria; 3 Department of Pediatrics and Adolescent Medicine, St. Anna Children’s Hospital, Medical University of Vienna, Waehringer Guertel 18-20, 1090 Vienna, Austria; 4 Department of Biomedical Imaging and Image-guided Therapy, Medical University of Vienna, Waehringer Guertel 18-20, 1090 Vienna, Austria

**Keywords:** Eosinophilia, Cystitis, Cystic mass, Pediatric, Dysuria, Bladder wall thickening

## Case summary

**Fig. 1:**
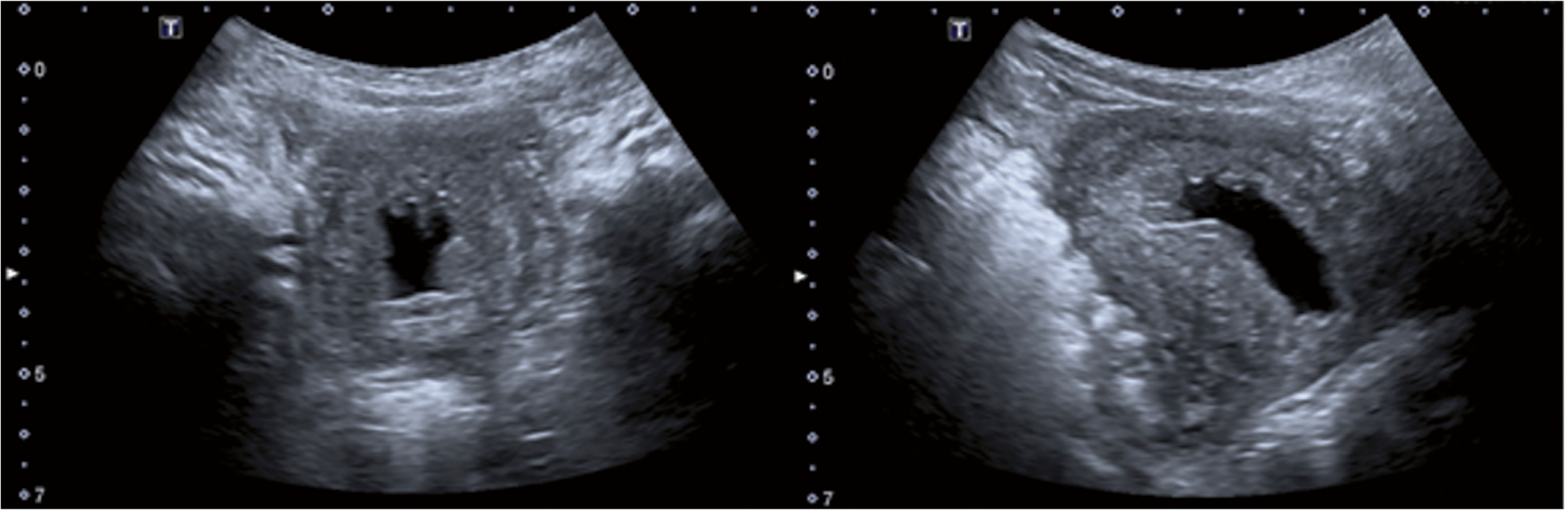
Sonography of the urinary bladder axial (left) and sagittal (right) displaying urinary bladder wall thickening (up to 2 cm) with heterogeneous echogenicity

A 3-year-old boy was admitted to our pediatric urology department for further investigation of dysuria and pollakisuria. He was previously healthy and throve well. Macroscopic and microscopic urinary assessment did not reveal any pathologic features. A sonography of the boy’s urinary tract revealed urinary bladder wall thickening (up to 2 cm) with heterogenous echogenicity (Fig. 1). Further imaging with magnetic resonance imaging scans unraveled these heterogenous wall thickenings as polypus endoluminal concavities. The only other abnormal finding upon routine examination was peripheral eosinophilia (28% of peripheral leukocytes and 2.47 × 10^9^/L absolute eosinophil count) in the boy’s blood count. Eosinophilia is defined as the presence of higher absolute and relative counts of eosinophils in the peripheral blood (> 5% of peripheral leukocytes and > 0.5 × 10^9^/L absolute eosinophil count) [1]. Causes for eosinophilia are highly diverse and include primary (clonal) forms caused by hematologic neoplasms, and the more common secondary (reactive) forms due to allergic disorders (e.g., asthma and atopic dermatitis), parasitic and fungal infections, rheumatological diseases (e.g., systemic lupus erythematosus and vasculitis), respiratory diseases (e.g., eosinophilic pneumonia), other neoplasms (e.g., solid tumors and lymphomas), dermatologic disorders (e.g., Wells syndrome), disorders of immune regulation (e.g., hyper IgE syndrome), gastrointestinal disorders (e.g., eosinophilic esophagitis), and drugs [2]. Through clinical, serological, urinary, and fecal examinations, we could not identify any secondary causes for eosinophilia.

**Fig. 2:**
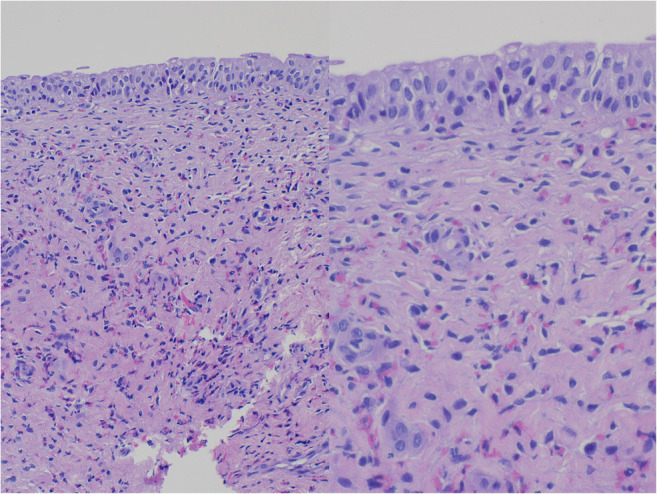
Histological specimen (hematoxylin and eosin stain, ×200 magnification [left] and ×400 magnification [right]) of our patient displaying edematous subepithelial tissue with partly accentuated perivascular eosinophilic inflammatory infiltrates of varying density with partly reactive widened urothelial tissue

For the first and most important step to rule out bladder malignancy (i.e., rhabdomyosarcoma), and to either diagnose or rule out collagen vascular diseases, the boy underwent cystoscopy and subsequent biopsy of the polypus endoluminal concavities. Histologic analysis described an inflammatory eosinophilic-dominated infiltrate with no signs of malignant disease (Fig. 2). Symptomatic treatment with oxybutynin (0.1 mg/kg three times a day) for the boy’s dysuria and pollakisuria was initiated. Initially, the boy’s symptoms were relieved, and the urinary bladder wall thickening declined after 1 month of treatment from 2 to 0.27 cm. However, about 3 months after diagnosis, urinary bladder wall thickening increased to 0.5 cm. The boy also experienced constipation, a common side effect of treatment with oxybutynin, and initial symptoms returned, with peripheral eosinophilia still at high levels (18% of peripheral leukocytes and 1.9 × 10^9^/L absolute eosinophil count). At the end of the fourth month of oxybutynin treatment, bladder wall thickening increased up to 1.2 cm and the boy presented with weight loss of 2.3 kg over a period of 4 months, abdominal distention, and markedly decreased subcutaneous fat. Laboratory tests revealed hypoalbuminemia and deficiencies of iron, vitamin D, and vitamin A.

To rule out other reasons for the weight loss, we performed further analysis which revealed IgA antibodies against transglutaminase 2 of more than ten times the upper reference value (> 200 U/mL) and positivity for endomysial antibodies (1:640). Together with positivity for endomysial antibodies (1:640) and the at-risk HLA-DQ2: A1*0505, B1*0202 genotype, the diagnosis of celiac disease was established [3].

**Questions**

1. What is the diagnosis and what is its most likely etiology?
2. What further examinations should be performed?
3. What is the treatment for this disease?
